# Application of Operating Room Nursing Intervention to Incision Infection of Patients Undergoing Gastrointestinal Surgery Can Reduce Complications and Improve Gastrointestinal Function

**DOI:** 10.3389/fsurg.2022.842309

**Published:** 2022-02-15

**Authors:** Ping Wang, Hong Chen, Qi Ji

**Affiliations:** Operating Room, The First People's Hospital of Lianyungang, Lianyungang, China

**Keywords:** gastrointestinal surgery, operating room nursing, incision infection, gastrointestinal function, complication

## Abstract

**Objective:**

To observe the influence of nursing intervention in operation rooms on incision infection of patients undergoing gastrointestinal surgery and the improvement of gastrointestinal function.

**Methods:**

A total of 340 patients who underwent gastrointestinal surgery in our hospital from June 2020 to August 2021 were included. According to the random number table, they were divided into the conventional nursing group (*n* = 170) and the operating room nursing group (*n* = 170). The conventional nursing group was treated with routine nursing intervention, while the operating room nursing group was treated with operating room nursing intervention. The incision infection, healing, gastrointestinal function recovery, and complications in the two groups were compared, and the patient care satisfaction was recorded.

**Results:**

The incidence of incision swelling, pain, and incision secretion in the operating room nursing group was significantly lower than that in the conventional nursing group (*p* < 0.05). The patients in the operating room nursing group had higher grade A healing than in the conventional nursing group, and lower grade B and grade C healing than in the conventional nursing group (*p* < 0.05). The time of anal exhaust, first defecation, and the time of gastric tube removal in the operating room nursing group were lower than those in the conventional nursing group (*p* < 0.05). The incidence of postoperative complications, such as incision infection, incision dehiscence, early inflammatory bowel adhesion, and abdominal abscess, in the operating room nursing group was lower than that in the conventional nursing group (*p* < 0.05). The total satisfaction degree in the operating room nursing group was significantly higher than that in the conventional nursing group (*p* < 0.05).

**Conclusion:**

Nursing intervention in operation room can reduce complications and improve gastrointestinal function when applied to patients undergoing gastrointestinal surgery due to incision infection.

## Introduction

It is easy to breed bacteria in the gastrointestinal tract. Therefore, patients undergoing gastrointestinal surgery are prone to infection of surgical incision and various other complications. Incision infection accounts for 15–20%, a high proportion, of nosocomial infections (1, 2). Two to four days after gastrointestinal surgery, the incision pain and body temperature of a patient will gradually return to normal. If the incision pain is not relieved or if there are signs, such as swelling and fever in the local incision, it indicates that a degree of infection might be present (3). The dehiscence and exudation that occur after the infection of an incision prolongs its healing time. If not treated in time, the infection may worsen and may eventually lead to organ dysfunction or systemic infection and death in severe cases (4, 5).

Although the operating room is an important place for clinical rescue, it is also a department with a high infection rate. Postoperative infection has always been a clinically difficult problem (6, 7). Literature shows that nursing interventions for patients undergoing gastrointestinal surgery can effectively reduce postoperative infection rate. To further reduce the infection rate of incisions after gastrointestinal surgery, we should not only standardize the disinfection and sterilization for the operation, but also do a good job of nursing in the operating room so as to reduce the infection rate of incision, improve the operation efficiency, and reduce the economic and psychological burden of patients (8). This study discusses the influence of nursing intervention in operating rooms on wound healing and gastrointestinal peristalsis of patients undergoing gastrointestinal surgery. The results are reported as follows.

## Data and Methods

### General Information

A total of 340 patients who underwent gastrointestinal surgery in our hospital from June 2020 to August 2021 were included in the study. They were divided into the conventional nursing group (170 patients) and the operating room nursing group (170 patients) according to the random number table. The inclusion criteria were as follows: patients undergoing elective surgery; patients who have no immune or endocrine disorders; patients with a certain have primary school education or above and can cooperate with the research. The exclusion criteria were as follows: patients with severe hepatic and renal insufficiency; patients with malignant tumor/s or mental diseases; patients with with coagulation disorders, i.e., anemia; patients who have cultural taboos on nursing measures; patients who have had failed treatment or operation and may prolong their stay in hospital; palliative surgery patients. This study was approved by the Hospital Ethics Committee, and informed consent was obtained from patients and their families.

### Research Methods

Routine nursing intervention was adopted in the conventional nursing group. Nurses in this group were tasked with the following: (1) timely update of the operating room equipment level; (2) conduct regular spot checks for health care personnel infection prevention awareness and measures to understand the situation, for understanding the improper health care personnel should be strengthened education; (3) the operating room head nurse actively cooperate with infection department work, responsible for the daily infection prevention and control management; (4) for the medical staff in and out of the operating room for infection prevention measures supervision.

The operating room nursing group adopted the nursing intervention in the operation room. Before the intervention measures are implemented, a management system and process and areward and punishment system was made to encourage staff to supervise each other. In the process of implementation, staff were tasked to study and check each other regularly to correct the deficiencies. The specific measures are divided into three parts: preoperative preparation, strengthening the management of the operating room, and meticulous care of the incision. The specific measures are as follows:

(2) Pre-operation: after receiving the notice of operation, the nurses in the operating room should have carefully checked the case data of the patient and understand their condition. The purpose, method, precautions, and expected results of operation should be introduced to the patient and their family. Nurses should have patiently solved the problems raised by the patient and their family. In addition, they should have fully understood the psychological state of the patient and psychologically guide their bad emotions and enhance their confidence in treatment. At the same time, the operating room nurses should have given preoperative instructions, telling the patient that they should strictly fast from food and water 8 h before operation to prevent gastrointestinal decompression. Lastly, they must assist clinicians to complete various examinations, inspecting and checking instruments and items required for surgery.
(3) Strengthening the management of the operation room: Nurses were tasked to manage the access of personnel in the operation room. Non-emergency operations were reasonably sequenced. The interval between two operations was more than 35 min, during which the operating room environment and air were disinfected. Thirty minutes before surgery, the operating room nurses should clean and disinfect the environment and tools in the operating room. The storage and management of sterile instruments should be managed by special personnel. Before entering the room, they should strictly strengthen aseptic operation. Hand washing was conducted according to the six-step washing method. The nurse in charge of the operation room conducted a hand hygiene check on the medical staff involved in the operation. Surgeons can only stay in the sterile area of the operating room. The gloves used in the operation shall be replaced immediately if they are torn or punctured by a sharp instrument. Used instruments shall not be reused. The temperature and humidity in the operation room were controlled within an appropriate range, and the thermal measures were maintained for patients to avoid incision infection caused by too low temperature.
(4) Incision care: Before the operation, the local skin at the incision site was disinfected. The disinfected area should cover 15–20 cm around the incision before a 3 m skin protective film is stuck on the incision. During the operation, after the abdominal cavity is opened, the incision should be protected by a full-layer protector in time to prevent bacteria or feces from polluting the incision. After applying a protective pad around the incision, the gastrointestinal tract can be cut. After the peritoneal suture was completed, the skin, subcutaneous tissue, and grass-roots layer of the incision in the patient were rinsed with 37°C normal saline. Afterwhich, the normal saline was wiped clean with gauze (the tumor flushing fluid is sterilized water for injection at 38–43°C, and the operation field is soaked for 3–5 min for 2–3 times), and the incision was sutured layer by layer. A dress with good adsorbability was applied to that incision site to avoid incision exudation.

### Observation Indicators

The wound infection and healing of two groups of patients were compared. Assessment of wound healing included: Grade A healing (wound healing is good without any adverse healing), Grade B healing (inflammation such as swelling, hematoma or effusion after wound healing), and Grade C healing (wound purulent needs dressing change).

The recovery of gastrointestinal function and the incidence of complications were compared between two groups. The nurse should accurately record the gastrointestinal function indicators of patients (including anal exhaust time, first defecation time and gastric tube removal time), along with complications (including incision infection, incision dehiscence, early inflammatory bowel adhesion, and peritoneal abscess), and calculate the incidence rate.

The satisfaction of patients with nursing care in the two groups was recorded. According to the actual situation of the operating room, a nursing satisfaction score scale was set up, which mainly included the service attitude, nursing operation, operation process, health education, nursing results, and other aspects of the medical staff. There were three grades, namely, very satisfactory (score >89), satisfactory (score: 60–89 points), and dissatisfied (score <60 points). The content reliability index of nursing satisfaction rating scale was 0.79, and the α coefficient of Kehlenbach was 0.784, which had good reliability.

### Statistical Methods

SPSS22.0 software was used for processing. The measurement of the experimental data were expressed as mean SD (x ± s), and the enumeration data were expressed in percent (%). *T*-test analysis was used for pairwise comparison of measurement data among groups, and the count data were tested by χ*2* test. The test level was α = 0.05, and a value of *p* < 0.05 indicated that the difference was statistically significant.

## Results

### Comparison of General Data Between the Two Groups

There was no significant difference in general information such as gender, age, and educational background between the two groups (*p* > 0.05) as shown in Table 1.

**Table 1:** Comparison of general data of patients between the two groups.

**Group**	**Gender**		**Educational background**		
	**Male**	**Female**	**Junior secondary and below**	**Senior high school**	**Bachelor degree or above**
Conventional nursing group (*n* = 170)	81	89	61	68	41
Operating room nursing group (*n* = 170)	83	87	65	67	38
*χ^2^ value*	0.047		0.248		
*P value*	0.828		0.883		
Group	Age (years)	Surgical type			
		Partial gastrectomy	Clearance of intestinal adhesions	Repair of duodenal perforation	Radical gastrectomy for gastric cancer
Conventional nursing group (*n* = 170)	41.82 ± 6.53	49	29	51	41
Operating room nursing group (*n* = 170)	42.59 ± 5.91	48	24	54	44
*t/χ^2^ value*	1.140	1.111			
*P value*	0.255	0.774			

### Comparison of Incision Infection Between the Two Groups

**Figure 1:**
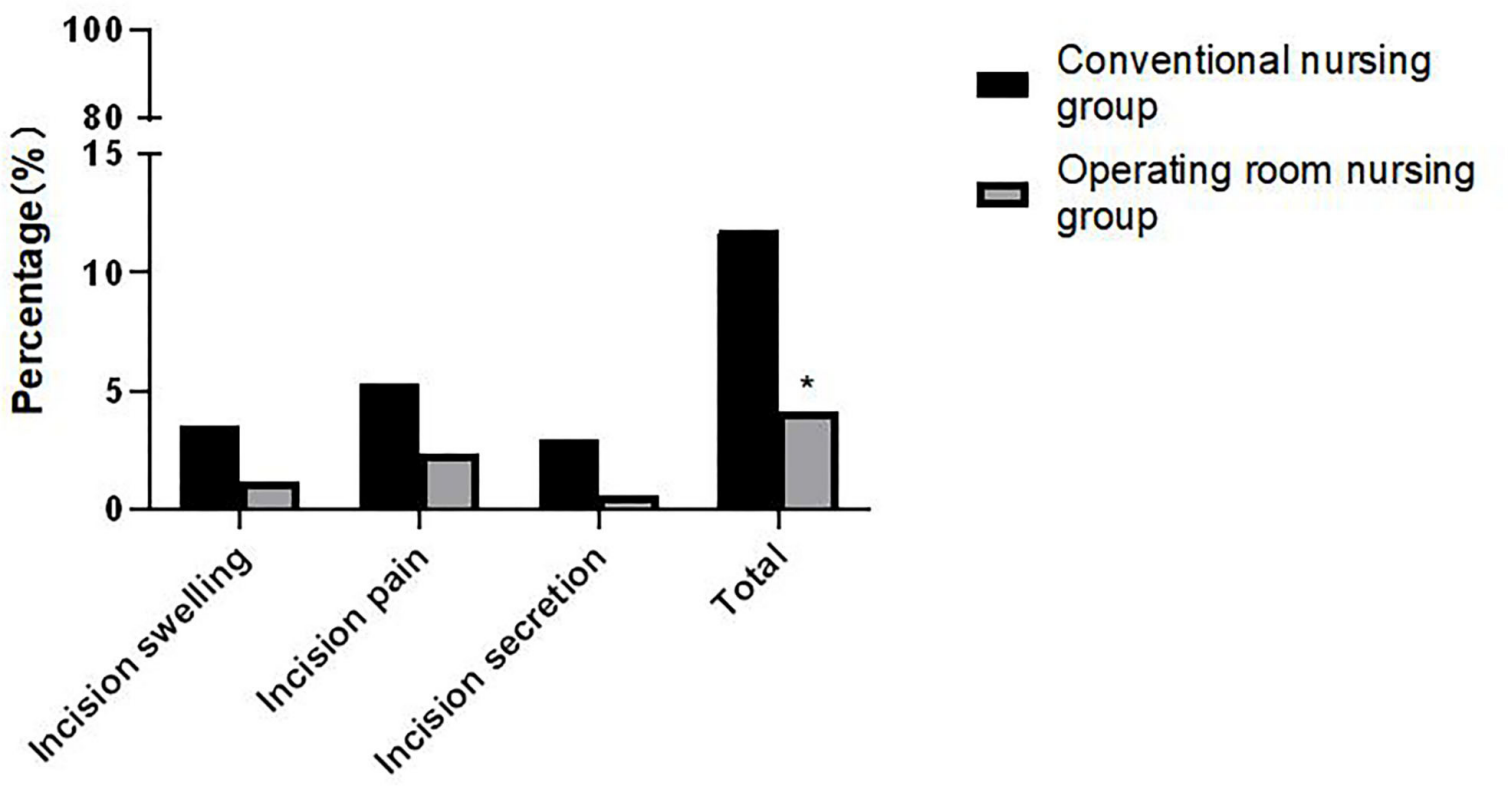
Comparison of incision infection between the two groups. Note: Compared with the conventional nursing group, *p < 0.05.

The incidence of incision swelling, pain, and incision secretion in the operating room nursing group was significantly lower than in the conventional nursing group. The differences were statistically significant (*p* < 0.05) as shown in Figure 1.

### Comparison of Wound Healing Between the Two Groups

**Figure 2:**
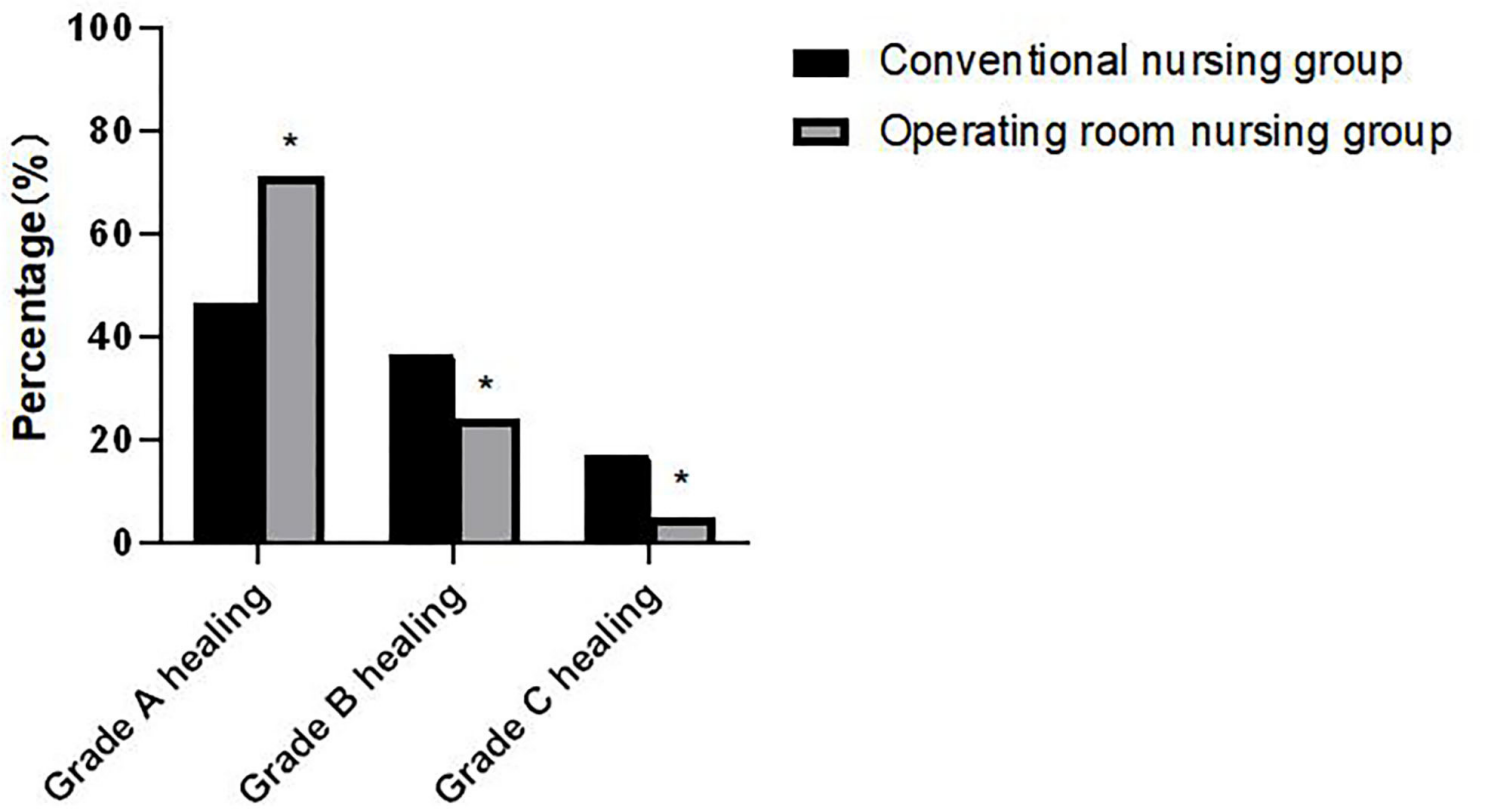
Comparison of wound healing between the two groups. Compared with the conventional nursing group, *p < 0.05.

The grade A healing rate of patients in the operating room nursing group was higher than that of the conventional nursing group, and the grade B and grade C healing rates were lower than those of the conventional nursing group. The differences were statistically significant (*p* < 0.05) as shown in Figure 2.

### Comparison of Gastrointestinal Function-Related Indicators Between the Two Groups

**Figure 3:**
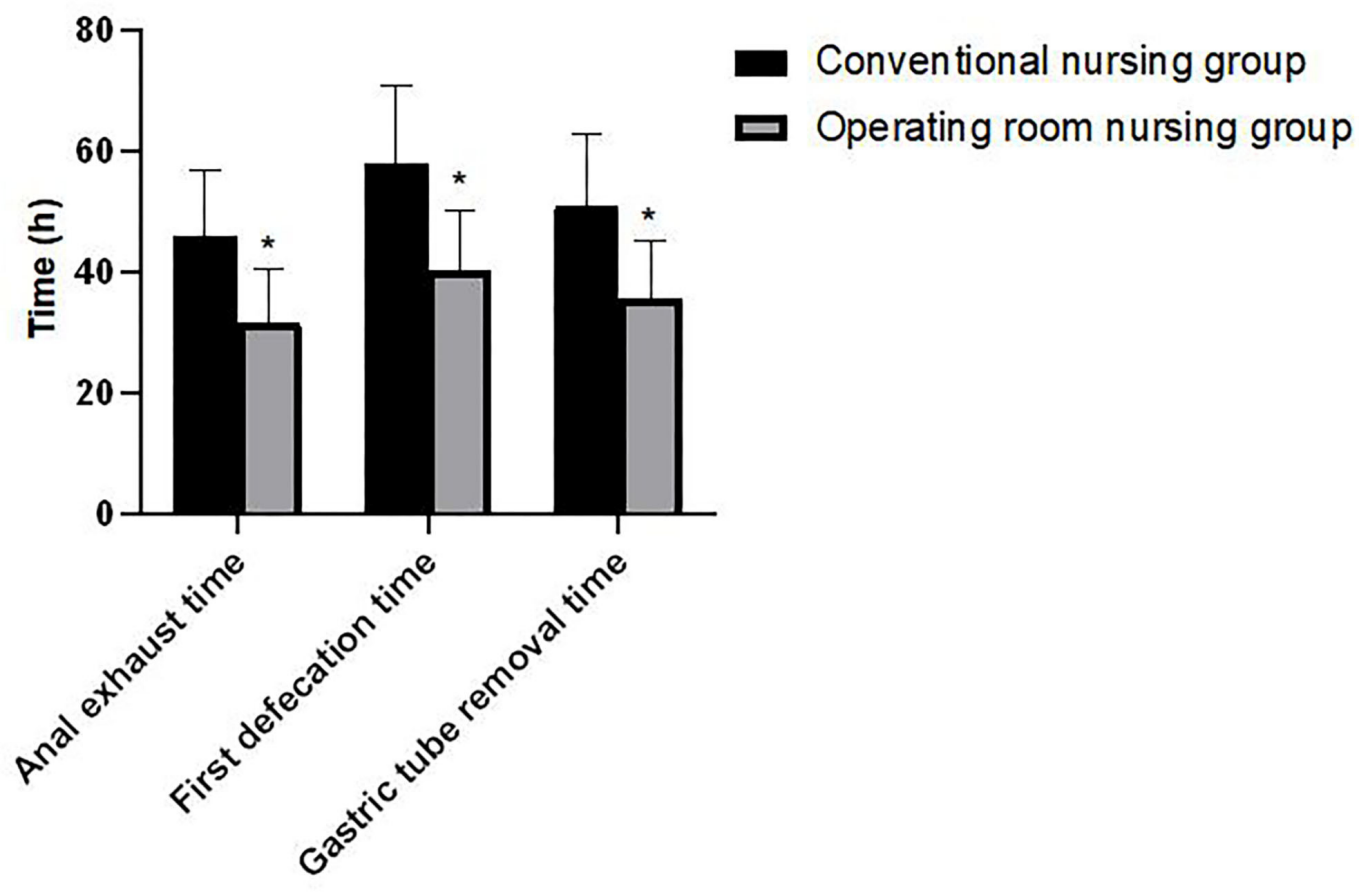
Comparison of gastrointestinal function-related indicators between the two groups. Compared with the conventional nursing group, *p < 0.05.

Patients in the operating room nursing group had lower anal exhaust time, first defecation time, and gastric tube removal time than those in the conventional nursing group. The differences were statistically significant (*p* < 0.05) as shown in Figure 3.

### Comparison of Postoperative Complications Between the Two Groups

**Figure 4:**
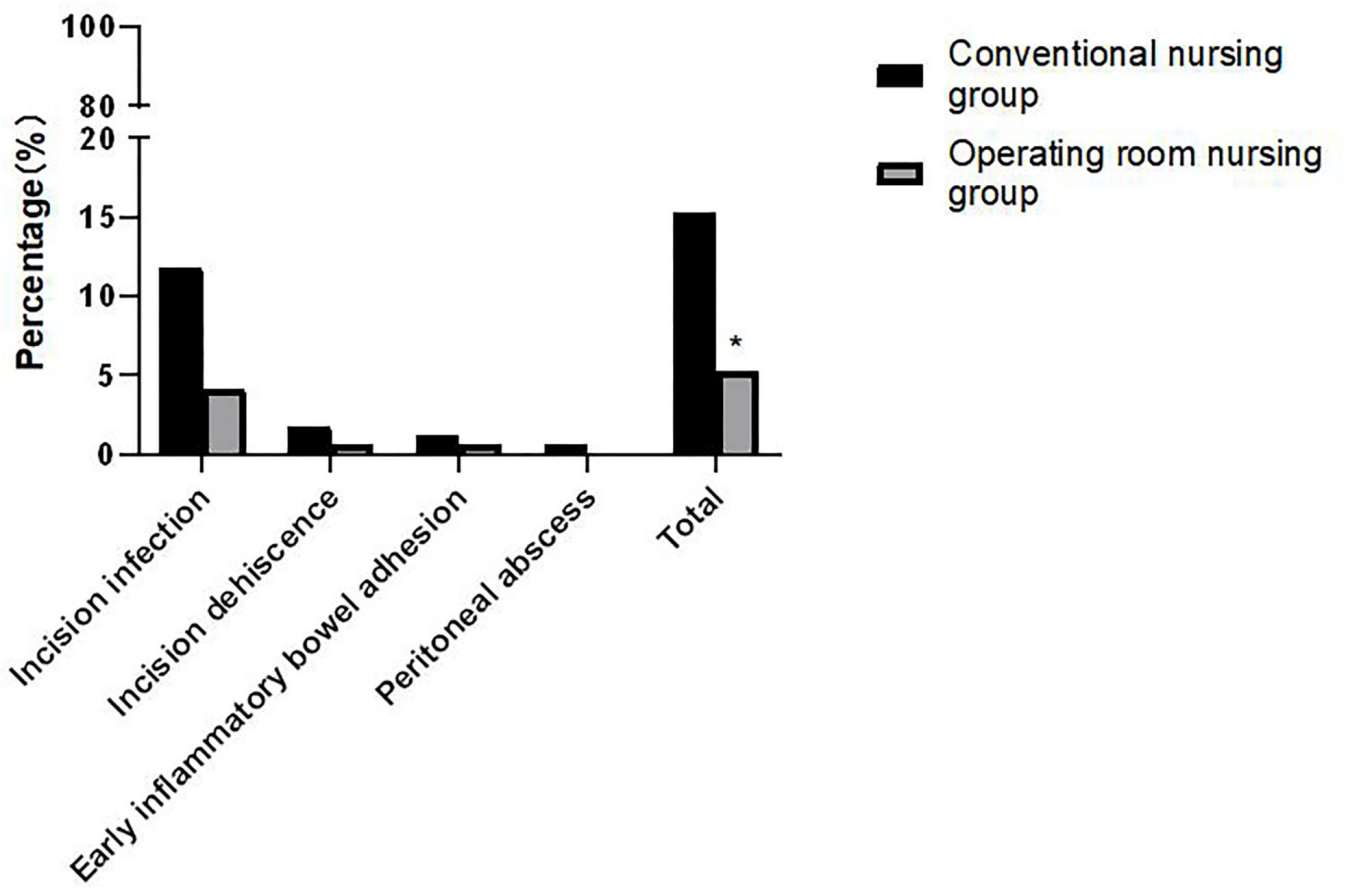
Comparison of postoperative complications between the two groups. Compared with the conventional nursing group, *p < 0.05.

The incidence of postoperative complications, such as incision infection, incision dehiscence, early inflammatory bowel adhesion, and peritoneal abscess, in the operating room nursing group was lower than that in the conventional nursing group. The difference was statistically significant (*p* < 0.05). As shown in Figure 4.

### Comparison of Nursing Satisfaction Between the Two Groups

**Figure 5:**
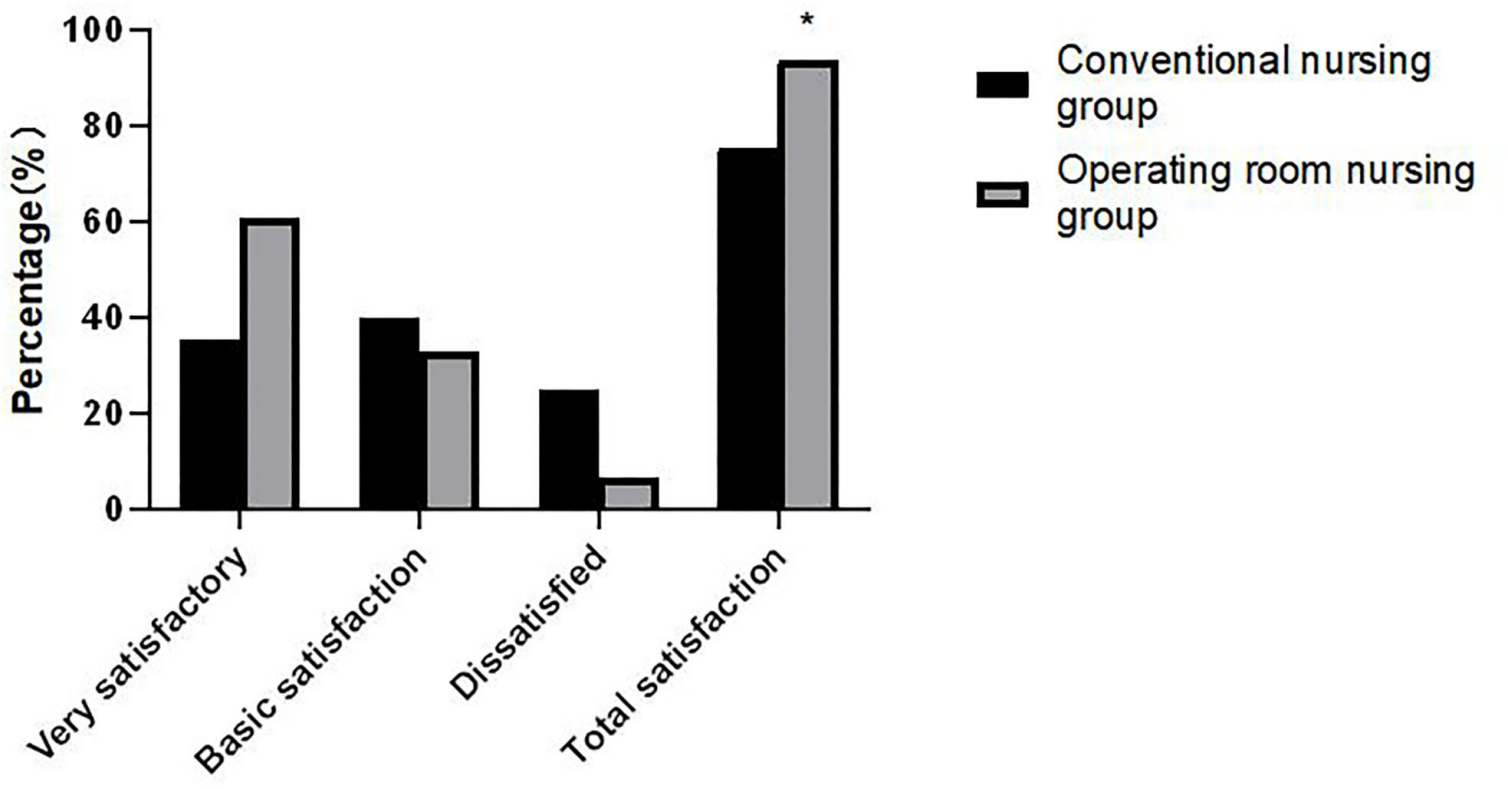
Comparison of nursing satisfaction between the two groups. Compared with the conventional nursing group, *p < 0.05.

The total satisfaction degree of the operating room nursing group (93.53%) was significantly higher than that of the conventional nursing group (75.29%). The difference was statistically significant (χ^2^ = 30.637, *p* < 0.001) as shown in Figure 5.

## Discussion

Postoperative infection of gastrointestinal incision is one of the common clinical complications at present. Studies have pointed out that the infection rate of gastrointestinal surgical incisions can be as high as 25%, which makes it a common complication in operating rooms (9). Compared with other types of surgery, gastrointestinal surgery has certain particularity. Gastrointestinal flora is abundant and most of the surgical incisions for gastrointestinal operations are of Class II and III. If the incision is not treated in time, the risk of surgical incision infection will increase to a certain extent, which could easily to aggravate the condition of the patient (10, 11). Incision infection is closely related to the operating room environment, the personnel involved in the operation, and the surgical instruments. Therefore, nursing in operation room plays an important role in patient safety.

The routine nursing mode in operating rooms has a certain effect on preventing infection. However, there are still many patients with different degrees of incision infection after operation. This seriously affects the treatment quality and work efficiency and thereby increases the incidence of medical disputes (12, 13). Therefore, finding suitable operating room nursing measures that can reduce complications, such as incision infection, is the focus of the current clinical research. In this study, the incidence of incision infection in the operating room nursing group was significantly lower than in the conventional nursing group. Wound healing was also significantly better than in the conventional nursing group. It indicated that nursing in operation room had clear advantages for reducing incision infection and promoting the recovery of patients.

According to the characteristics of gastrointestinal surgery, nursing in operating room should strictly make preparation for the operation, including checking the case data of the patient, grasping the basic situation of the patient, and preparing the skin, which can reduce the chance of bacterial infection to some extent. Secondly, the whole nursing process was centered on strengthening aseptic operation, strict access control in operation rooms, and reducing the risk of cross-infection. Medical staff involved in the operation should strictly wash their hands according to the six-step washing method to ensure hand hygiene, ensure disinfection in the operation area, ensure the operating room environmental health, and reduce bacterial adhesion. Staff should also strictly control the flow of operating room staff and the number of surgical visitors to reduce the chance of airborne bacteria. Finally, they are to cooperate with surgeons during incision processing, disinfection of local skin incision, taking the full layer protector to protect treatment in time, and, after the completion of the peritoneal suture, clean the incision using normal saline irrigation in order to block the path of bacterial infection, and avoid infection (14–16).

Gastrointestinal surgery can arouse the excitement and stimulation of the sympathetic nervous system of the gastrointestinal tract of patients. At the same time, due to the stimulation of anesthetics, traction and other factors, patients are very likely to suffer from inhibition of gastrointestinal peristalsis after surgery, which in turn leads to symptoms of gastrointestinal dysfunction, such as non-flatus, non-defecation and abdominal distension. This may affect the surgical effect and rehabilitation process of patients (17–19). This study showed that the patients in the operating room nursing group had lower anal exhaust time, first defecation time, and gastric tube removal time than those in the conventional nursing group. It is proved that nursing in operating room was superior to the routine nursing in promoting the recovery of gastrointestinal function. The smooth recovery of gastrointestinal function is an important symbol for the success of the operation. If gastrointestinal dysfunction occurs, it will not only cause water and electrolyte disorders, but also other complications, such as incision infection, dehiscence, early inflammatory bowel adhesion, abdominal abscess, and, in severe cases, death (20, 21). In this study, the incidence of postoperative complications in the operating room nursing group was lower than in the conventional nursing group. Take various preventive measures to reduce the injury caused by operation and the infection rate of incision, further promote the recovery of patients' gastrointestinal function and reduce complications.

Nursing in the operating room gives comfort and encouragement to the psychology of patients. It also introduces successful operations, which may reduce patient anxiety toward similar procedures. By extension, it can improve the satisfaction of patients with the nursing service (22). The results of this study demonstrated that the total satisfaction in the operating room nursing group was significantly higher than that in the conventional nursing group.

To sum up, nursing interventions in the operating room for patients undergoing gastrointestinal surgery can effectively reduce the infection rate of incisions, promote incision healing, improve the gastrointestinal function of patients, and reduce postoperative complications.

## Data Availability Statement

The original contributions presented in the study are included in the article/supplementary material, further inquiries can be directed to the corresponding author.

## Ethics Statement

The studies involving human participants were reviewed and approved by the Ethics Committee of the First People's Hospital of Lianyungang. The patients/participants provided their written informed consent to participate in this study.

## Author Contributions

PW was responsible for the writing of the manuscript. HC was responsible for the design of the study. QJ was mainly responsible for the guidance. All authors contributed to the article and approved the submitted version.

## Conflict of Interest

The authors declare that the research was conducted in the absence of any commercial or financial relationships that could be construed as a potential conflict of interest.

## Publisher's Note

All claims expressed in this article are solely those of the authors and do not necessarily represent those of their affiliated organizations, or those of the publisher, the editors and the reviewers. Any product that may be evaluated in this article, or claim that may be made by its manufacturer, is not guaranteed or endorsed by the publisher.
